# Skewed Cellular Distribution and Low Activation of Functional T-Cell Responses in SARS-CoV-2 Non-Seroconvertors

**DOI:** 10.3389/fimmu.2022.815041

**Published:** 2022-05-10

**Authors:** Athina Kilpeläinen, Esther Jimenez-Moyano, Oscar Blanch-Lombarte, Dan Ouchi, Ruth Peña, Bibiana Quirant-Sanchez, Raul Perez-Caballero, Anna Chamorro, Ignacio Blanco, Eva Martínez-Caceres, Roger Paredes, Lourdes Mateu, Jorge Carrillo, Julià Blanco, Christian Brander, Marta Massanella, Bonaventura Clotet, Julia G. Prado

**Affiliations:** ^1^ IrsiCaixa AIDS Research Institute, Badalona, Spain; ^2^ Germans Trias i Pujol Research Institute (IGTP), Badalona, Spain; ^3^ CIBERINFEC, ISCIII, Madrid, Spain; ^4^ Department of Cell Biology, Physiology, Immunology, Universitat Autònoma de Barcelona, Cerdanyola del Vallès, Spain; ^5^ Immunology Department, Hospital Universitari Germans Trias i Pujol, Badalona, Spain; ^6^ Lluita contra la SIDA Foundation, Hospital Universitari Germans Trias i Pujol, Badalona, Spain; ^7^ Clinical Genetics and Genetic Counseling Program, Hospital Universitari Germans Trias i Pujol, Badalona, Spain; ^8^ ICREA, Barcelona, Spain; ^9^ Infectious Diseases Department, Hospital Universitari Germans Trias i Pujol, Badalona, Spain; ^10^ University of Vic–Central University of Catalonia (UVic-UCC), Vic, Catalonia, Spain

**Keywords:** SARS-CoV-2, non-seroconvertor, cellular immunity, T cell subsets, immune activation, function

## Abstract

The role of T cells in the control of SARS-CoV-2 infection has been underestimated in favor of neutralizing antibodies. However, cellular immunity is essential for long-term viral control and protection from disease severity. To understand T-cell immunity in the absence of antibody generation we focused on a group of SARS-CoV-2 Non-Seroconvertors (NSC) recovered from infection. We performed an immune comparative analysis of SARS-CoV-2 infected individuals stratified by the absence or presence of seroconversion and disease severity. We report high levels of total naïve and low effector CD8+ T cells in NSC. Moreover, reduced levels of T-cell activation monitored by PD-1 and activation-induced markers were observed in the context of functional SARS-CoV-2 T-cell responses. Longitudinal data indicate the stability of the NSC phenotype over three months of follow-up after infection. Together, these data characterized distinctive immunological traits in NSC including skewed cellular distribution, low activation and functional SARS-CoV-2 T-cell responses. This data highlights the value of T-cell immune monitoring in populations with low seroconversion rates in response to SARS-CoV-2 infection and vaccination.

## Introduction

The COVID-19 pandemic is caused by SARS-CoV-2, the newest coronavirus crossing into the human population. The pandemic accounts for millions of infected people and around 6 million deaths worldwide. Despite the scientific success in rapidly generating SARS-CoV-2 vaccines, the number of infected people and the burden on healthcare systems continues (1). Consequently, continuous characterization of the functional features of immune protection of SARS-CoV-2 infection are needed.

The disease outcome of SARS-CoV-2 infection is associated with a wide degree of interindividual heterogeneity. These divergences may be associated with the level of immunocompetence and the tight balance between immune control and immunopathogenesis after infection (2). Indeed, beneficial and detrimental aspects of the immune responses elicited by SARS-CoV-2 infection have been described. As detrimental aspects, secondary multi-organ complications and persistent symptomatology for months in approximately 10% of the total infected people or “long COVID” (3) have been associated with the persistence of inflammatory profiles and tissue damage. As beneficial aspects, cellular and humoral immune responses have been linked to protection from disease severity against other coronaviruses such as SARS-CoV-1 (4) and the induction of B and T cell memory responses has been described in most of the individuals recovered from SARS-CoV-2 infection (5).

Cellular perturbations and immune profiles have been related to disease severity (6–8). Moreover, disease outcome have been correlated with early signatures of soluble meditators including growth factors, type-2/3 cytokines, type-1/2/3 cytokines, and chemokines in SARS-CoV-2 infection (9). High levels of cytokine and chemokine production upon infection, the so-called cytokine storm has been observed in SARS, MERS and SARS-CoV-2 infections, potentially leading to severe tissue damage (10). Patients suffering from severe COVID-19-induced acute respiratory distress syndrome have been described to display elevated levels of Interleukin-6 (IL-6), lymphopenia with low counts of CD8+ T cells, natural killer (NK) and naïve T helper cells, while B cells remained mainly unaffected (11). Along with increased IL-6, elevated IL-10, IL-2 and IFN-γ peripheral blood levels have been described (12). Finally, higher plasma levels of IL2, IL7, IL10, GSCF, IP10, MCP1, MIP1A, and TNFα were observed in patients admitted to the ICU (13). On the other hand, neutralizing antibodies have been shown to predict severity and survival (14). Frequencies of SARS-CoV-2-specific CD4+CD40L+ T cells and Spike-specific B cells have been associated with anti-SARS-CoV-2 antibodies and the magnitude of neutralizing activity in children (15). However, high neutralizing antibody titers do not positively correlate with less severe clinical outcomes (16, 17).

Multiple studies support the relevance of cellular immunity in the control and prevention of SARS-CoV-2 infection. Antiviral CD4+ and CD8+ T-cell responses are key in the control of viral infections (18–20). Data from SARS-CoV-2 animal models support the role of T-cell immunity as a correlate of protection from infection (21) and virus-specific CD4+ and CD8+ T-cell responses are considered key players in the resolution and long-term protection from infection (22). The presence of SARS-CoV-2 CD4+ and CD8+ T-cell responses up to 6 months after infection in mild to moderate clinical course and persistent immunological alterations in the memory compartment have been described after SARS-CoV-2 infection (5, 23). Moreover, protection through pre-existent SARS-CoV-2 cross-reactive CD4+ T cell responses to other coronaviruses has been proposed as a mechanism limiting disease severity in a fraction of individuals (24, 25).

Phenotypic and functional characterization of cellular immunity against SARS-CoV-2 in the absence of immune pathogenesis is essential to identify the determinants of immune protection from infection and disease severity. To gain deeper insights into the characterization of cellular immunity in the absence of pathogenesis and serconversion, we focused on a group of SARS-CoV-2 Non-Seroconvertors (NSC) and compared their cellular immunity to individuals who seroconverted. Lack of seroconversion in SARS-CoV-2 infection has been demonstrated in 2-17% of Convalescent individuals (25–28) and cellular responses have been observed in 41 to 78% of these individuals (25, 27, 29). Currently, the functional characterization of T cell responses in NSC remains limited due to the low numbers of individuals identified, lack of confirmation of SARS-CoV-2 infection by PCR, or specific focus in CD8+ T cells. Functional T-cell responses was recently described in a small number of seronegative convalescent COVID-19 patients (30). To gain more knowledge on this subset of individuals, we characterized SARS-CoV-2 T cell immunity in NSC in terms of the cellular landscape, activation and functional profile and compared to recovered individuals that seroconverted stratified by SARS-CoV-2 disease severity. Our analyses identify differential immunological traits in NSC. We observed skewed CD8+ T-cell distribution towards an increase in naïve populations in the absence of overt activation of functional SARS-CoV-2-specific T cell responses. Our current analyses provide additional information on the characterization of T-cell immunity in the absence of SARS-CoV-2 seroconversion giving further insights of the contribution T cells to viral control. Moreover, our data highlight the importance to monitor T-cell immunity in those populations with low levels of antibodies in response to SARS-CoV-2 infection and vaccination.

## Materials and Methods

### Study Participants

**Figure 1:**
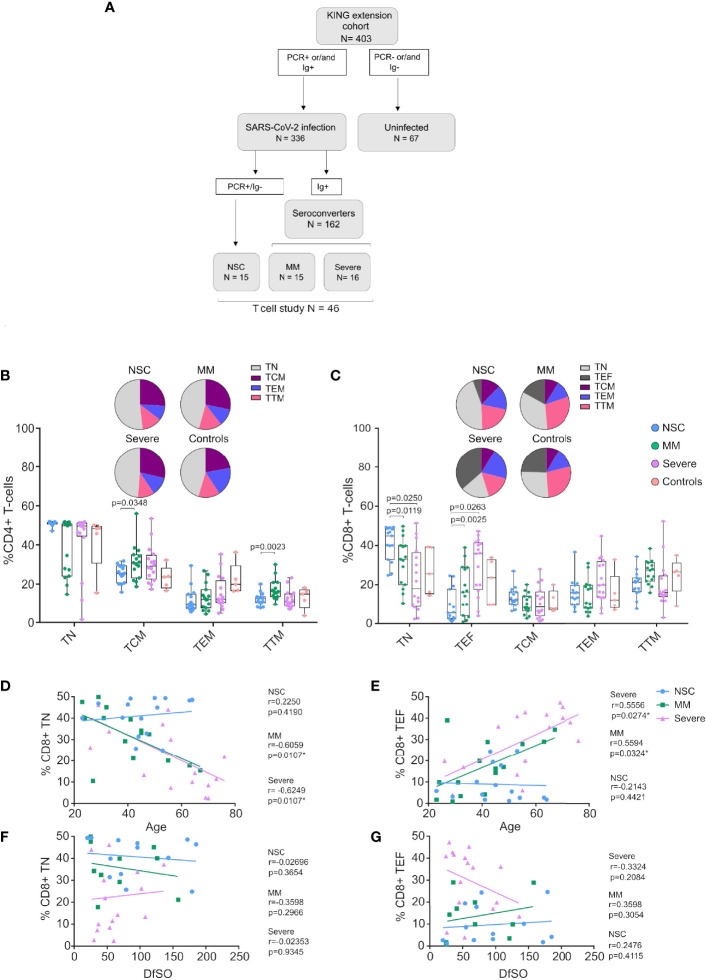
CD4+ and CD8+ T-cell subset distribution in Non-Seroconvertors. **(A)** Flow chart of the study groups, including Non-Seroconvertors (NSC), Mild-moderate (MM), Severe **(B, C)** Cryopreserved PBMCs were cultured and surface stained with antibodies targeting CD4 and CD8 as well as T-cell lineage markers CD45RA, CCR7, CD27 to distinguish T-central memory cells (TCM), T effector memory cells (TEM), T transitional memory cells (TTM), T Naïve cells (TN) and T effector cells (TEF). Cells were pre-gated for lymphocytes and live cells. Whisker plots show the frequencies of CD4+ T-cell subsets and pie charts represent the frequency distribution of CD4+ and CD8+ T-cell subsets in each group (Panel **B, C**, respectively). Statistical analyses were performed using a multivariable regression analysis. Only significant values are indicated in the Figure. Linear regression was performed plotting relative frequencies of CD8+ TN **(D)** and CD8+ TEF **(E)** against the age of the study participants, as well as relative frequencies of CD8+ TN **(F)** and CD8+ TEF **(G)** against the Days from Symptom Onset (DfSO) to sample. Correlation analyses were performed using Spearman’s rank correlation test; r- and p-values are reported for each group.

The KING extension cohort (N=403) is composed of 336 SARS-CoV-2 infected individuals, including 120 individuals who required hospitalization. For the present study, we focused on individuals with a SARS-CoV-2 infection diagnosis by PCR without seroconversion (Non-Seroconverters, NSC, N=15), and compared NSC with two groups of seroconvertors stratified by disease severity (Mild-moderate, MM, N=15 and Severe, N=16) (**Figure 1A**). The study groups were selected from the first wave of the COVID-19 pandemic in Spain. In brief, the NSC were defined as SARS-CoV-2 infected individuals by PCR diagnosis with no detectable SARS-CoV-2 IgG, IgA and IgM by two independent ELISAs (in-house and a commercially available ELISA). All NSC displayed asymptomatic or symptomatic infection not requiring hospitalization. NSC represented 4.5% of the infected KING extension cohort. The first serology test was obtained a median of 63 Days from Symptom Onset (DfSO) and lack of seroconversion was confirmed a median of 94.5 DfSO. The MM were defined as SARS-CoV-2 infected individuals diagnosed by PCR and/or SARS-CoV-2 IgG ELISA with asymptomatic or symptomatic infection not requiring hospitalization. The Severe were defined as SARS-CoV-2 infected individuals diagnosed by PCR and/or SARS-CoV-2 IgG ELISA with symptomatic infection requiring hospitalization. The first serology test was obtained a median of 38.5 and 24 days DfSO in MM and Severe, respectively. The clinical characteristics of the study groups are summarized in **Table 1**. Biological samples were available for all study groups and additional longitudinal samples were available for 6 NSC a median of 93 DfSO. Also, we obtained samples from controls from the Catalan Blood and Tissue Bank before 2019 as methodological controls (controls, N=5). The information regarding age, sex and other epidemiological data was not available for controls because the Catalan Blood and Tissue Bank did not provide it. None of the study participants had received a COVID-19 vaccine during the course of the study.

**Table 1:** Clinical characteristics of the study groups.

	NSC	MM	Severe		p-value^4^	
	(n=15)	(n=15)	(n=16)	NSC vs MM	NSC vs Severe	MM vs Severe
Sex, Female, n (%)	14 (88)	11 (69)	4 (27)	0.801	0.005*	0.137
Age at diagnosis of SARS-CoV-2 median, [IQR^1^]	45 [40-53]	41 [29-48]	56 [48-70]	>0.999	0.108	0.009*
Days from positive PCR to sample, [IQR]	66 [43-130]	57 [21-173]	44 [30-85]	>0.999	0.742	>0.999
DfSO to first positive PCR, [IQR]	6.5 [1-12.7]	7 [2-89]	8 [5-12]	>0.999	>0.999	>0.999
DfSO to sample, [IQR]	79 [54.5-157.5]	55 [28.7-122.3]	53.5 [35.5-88.5]	0.590	0.272	>0.999
DfSO to second sample, [IQR]	92.5 [82.5-110.8]					
Confirmed Positive PCR, n (%)	16 (100)	7 (47)	7 (47)			
Positive serology, n (%)	0 (0)	15 (100)	16 (100)	<0.0001*	<0.0001*	>0.9999
Plasma neutralizing titer, reciprocal dilution median, [IQR]	<60^2^	258 [60-378]	1276 [557-3158]	0.011*	<0.0001*	0.010*
Severe, n (%)^3^	0 (0)	0 (0)	16 (100)	>0.999	<0.0001*	<0.0001*
Asymptomatic, n (%)	2 (13)	2 (13)	0 (0)	>0.999	0.578	0.578
Co-morbidities, any comorbidity, n (%)	10 (67)	6 (40)	12 (75)	>0.999	0.012*	0.084
HTA	1 (7)	0 (0)	6 (38)			
Obesity	0 (0)	0 (0)	6 (38)			
Allergies	3 (20)	4 (27)	2 (13)			
Asthma	1 (7)	0 (0)	2 (13)			
Primary Immunodeficiency	1 (7)	0 (0)	0 (0)			
Autoimmunity	1 (7)	0 (0)	0 (0)			
Others	7 (47)	5 (33)	9 (56)			

NSC, Non-Seroconvertors; MM, Mild-moderate.

^1^[IQR]: inter-quartile range.

^2^<60, below the limit of detection.

^3^Severe presentation of SARS-CoV-2 requiring hospitalization.

^4^Analysis performed by non-parametric ANOVA comparing all groups.

^*^Statistical significance.

### Determination of Anti-SARS-CoV-2 Antibodies by Enzyme-Linked Immunosorbent Assays

The presence of anti-SARS-CoV-2 antibodies in serum or plasma samples was evaluated using two independent Enzyme-linked Immunosorbent Assays (ELISA). The first was an in-house developed sandwich-ELISA. Briefly, Nunc MaxiSorp ELISA plates were coated overnight at 4°C with 50 ml of capture antibody (anti-6xHis antibody, clone HIS.H8; ThermoFisher Scientific) at 2 mg/mL in PBS. After washing, plates were blocked for two hours at room temperature using PBS containing 1% of bovine serum albumin (BSA, Miltenyi biotech). 50 ml (1 mg/mL in blocking buffer) of the following SARS-CoV-2 derived antigens: S1+S2 subunits of the Spike (S) protein and receptor-binding domain (RBD, Sino Biological) were subsequently added and incubated overnight at 4°C. Each plasma sample was evaluated in duplicates at a 1/100 dilution in blocking buffer for each antigen. Antigen free wells were also assessed in parallel for each sample in the same plate to evaluate sample background. Serial dilutions of a positive plasma sample were used as standard. A pool of 10 SARS-CoV-2 negative plasma samples, collected before June 2019, were included as the negative control. Samples were assayed at 1/100 dilution in blocking buffer for one hour at room temperature. The following reagents were used as secondary antibodies: HRP conjugated (Fab)2 Goat anti-human IgG (Fc specific) (1/20000), Goat anti-human IgM (1/10000), and Goat anti-human IgA (alpha chain specific) (1/20000) (all from Jackson Immunoresearch). Secondary antibodies were incubated for 30 minutes at room temperature. After washing, plates were revealed using o-Phenylenediamine dihydrochloride (OPD) (Sigma Aldrich) and the enzymatic reaction was stopped with 4N of H2SO4 (Sigma Aldrich). The signal was analysed as the optical density (OD) at 492 nm with noise correction at 620 nm. The specific signal for each antigen was calculated after subtracting the background signal obtained for each sample in antigen-free wells. The second ELISA was a commercially available IgM and IgG class antibody ELISA against the SARS-CoV-2 NP (ImmunoDiagnostics, Hongkong). Briefly, serum samples were diluted at 1:100 and incubated for 1 hour at room temperature. Anti-NP antibodies were captured by immobilized NP recombinant protein. After incubation, captured antibodies were measured by an absorbance microplate reader at 450 nm. The antibody results were expressed as an index value, calculated as the ratio of the OD value for each sample to the OD value of the cut-off (0,200). The test was considered positive when the index value was ≥ 1.1, borderline when the index value was ≥ 0.9 to < 1.1, and negative when the index value was < 0.9.2.3 Pseudovirus neutralization assay.

HIV-1 reporter pseudoviruses expressing SARS-CoV-2 S protein and Luciferase were generated. pNL4-3.Luc.R-.E- was obtained from the NIH AIDS repository (Connor RI, Chen BK, 195). SARS-CoV-2.SctΔ19 was generated (Geneart) from the full SARS-CoV-2 S gene sequence with a deletion of the last 19 C-terminal codons (31), human-codon optimized and inserted into pcDNA3.4-TOPO. Expi293F cells were transfected using the Expifectamine Reagent (Thermo Fisher Scientific, Waltham, MA, USA) with pNL4-3.Luc.R-.E- and SARS-CoV-2.SctΔ19 at an 8:1 ratio, respectively. Control pseudoviruses were obtained by replacing the S protein expression plasmid with a VSV-G protein expression plasmid as reported previously (31). Supernatants were harvested 48 h after transfection, filtered at 0.45 µm, frozen and titrated on HEK293T cells overexpressing WT human ACE-2 (Integral Molecular, USA). For the neutralization assay, 200 TCID_50_ of pseudoviral supernatant was preincubated with serial dilutions of heat-inactivated serum or plasma samples (ranging from 1/60 to 1/14580) for 1h at 37°C and then added to ACE2-overexpressing HEK293T cells. After 48 h, cells were lysed with Britelite Plus Luciferase reagent (Perkin Elmer, Waltham, MA, USA), and luminescence was measured for 0.2 s with the EnSight Multimode Plate Reader (Perkin Elmer). Data were fitted to a four-parameter logistic curve with variable slope using Graph Pad Prism software (v8.3.0). IC50 values are expressed as reciprocal dilution.

### Immunophenotype of SARS-CoV-2 T-Cell Responses and Proliferation Assays

To measure T cells and SARS-CoV-2 specific T-cell responses, cryopreserved PBMCs were stimulated in the absence or presence of S and NP recombinant proteins (5 μg/mL, Sinobiological, China) using as control Staphylococcal enterotoxin B (SEB) (1 μg/mL, Sigma-Aldrich), with CD28/49d co-stimulatory molecules (1 μg/mL, BD) for 17 h at 37°C in a 5% CO_2_ incubator. After incubation, PBMCs were treated with Monensin A (1 μg/mL, BD Golgi STOP, Thermo Fisher Scientific) for 6h at 37°C and stored overnight at 4°C. The next day, cells were stained with a combination of T-cell lineage markers and functional markers for immunophenotyping. In brief, cells were labelled with a viability dye (APC-Cy7, Thermo Fisher Scientific) for 30 min at room temperature (RT) and surface stained for 30 min at RT with anti-human antibodies for CD3 (A700, clone UCHT1, BD), CD4 (FITC,clone OKT4, Biolegend), CD8 (V500, clone RPA-T8, BD), CD45RA (BV786, clone HI100, BD), CCR7 (PE-CF594, clone 150503, BD), CD27 (BV605, clone L128, BD), PD-1 (BV421, clone EH12.1, BD) and activation induced markers (AIM) CD25 (A647, clone BC96, Biolegend), OX40 (PE, clone Ber-ACT35, Biolegend) and CD137 (PeCy7 clone 41BB, Biolegend). Afterwards, cells were fixed with Fix/Perm Buffer A (Thermo Fisher Scientific) for 15 min at RT and stained intracellularly with Fix/Perm Buffer B and antibodies for TNF (PE-Cy7, clone MAb11, BioLegend), IFN-γ (BV711, clone B27, BD), and IL-2 (BV650, clone MQ1-17H12, BD) for 20 min at RT. Finally, cells were resuspended and fixed in formaldehyde 1% and acquired on LSR Fortessa cytometer using FACSDiVa software (BD). Data analysis was performed using FlowJo software version 10.0.7 (Tree Star, Ashland, OR, USA). Specific gates were defined using fluorescence minus one (FMO) controls (**Figure S1**). Surface markers were measured in total CD4+ and CD8+ T cells. The CD4+ and CD8+ T-cell subsets were differentiated based on CD27 and CCR7 expression: naïve (TN: CD45RA^+^, CD27^+^, CCR7^+^), effector (TEF: CD45RA^+^, CD27^-^, CCR7^-^, only for CD8+), central memory (TCM: CD45RA^-^, CD27^+^, CCR7^+^), transitional memory (TTM: CD45RA^-^, CD27^+^, CCR7^-^), and effector memory (TEM: CD45RA^-^, CD27^-^, CCR7^-^), as previously described (32, 33) (**Figures S2A, B**). To measure proliferation, cryopreserved PBMCs were labeled with CFSE, (Thermo Fisher –V12883, 0.5 μM) and cultured in the absence or presence of S and NP recombinant proteins (5 μg/mL, Sinobiological, China) and Staphylococcal enterotoxin B (SEB) (1 μg/mL, Sigma-Aldrich) as a positive control, for ten days at 37°C in a 5% CO_2_ incubator. After incubation, PBMCs were surface stained for 30 min at room temperature (RT) with anti-human antibodies for CD3 (A700, clone UCHT1, BD), CD4 (A647, clone RPA-T4, BD), and CD8 (V500, clone RPA-T8, BD). FlowJo 10.0.7 was used to analyze flow cytometric data. The frequency of T-cell cytokine responders against S and/or NP proteins were defined by the presence of at least one cytokine. In addition, the frequency of total T-cell responders against S and/or NP proteins were defined by the presence of at least one cytokine and/or AIM. We used a 0.2% cut-off value for positivity following background subtraction. To determine the SARS-CoV-2 specific T-cell proliferation, we subtracted background signal obtained from unstimulated cells.

Single-cell analysis were performed using the statistical package R (v3.6.3). Cells were compensated and selected based on its antigen expression (TNF, IL-2 and IFN-γ intensity) and normalized before performing UMAP dimensionality reduction. We used FlowJo 10.0.7 for the graphical representation of the single-cell analysis.

### Statistical Analyses

Descriptive and comparison tests were performed using Graph Pad Prism, version 6 (GraphPad Software, Inc., San Diego, CA, USA). Non-parametric ANOVA (Kruskal Wallis) tests were used for Groupwise comparisons and adjusted for multiple comparisons (Dunn’s test). Wilcoxon matched-pairs signed-rank tests were used to compare parameters between sampling time points. Spearman’s rank correlation coefficient test was used for correlation analysis. In the analysis of T-cell subsets, median values within phenotypes were normalized to 100%. A multivariable regression analysis was used to compare groups, adjusting for age, gender, days from symptom onset, comorbidities and comedications. The final model was obtained by means of a stepwise variable selection procedure. For each model, the estimator with its confidence interval (CI) of 95% was reported along with the p-value. The statistical package R (v3.6.3) was used for the analysis.

## Results

### Non-Seroconvertors Present Skewed T Cell Distribution Towards Higher Naïve and Lower Effector CD8+ T Cells

Previous studies have demonstrated alterations in immunological parameters following SARS-CoV-2 infection, including lymphopenia with a marked decrease in CD8+ T cells and alterations in the frequency of CD8+ T-cell subsets compared to healthy donors (8, 34). We determined T cell subsets distribution in NSC by staining for CD3, CD4 and CD8 markers and differentiation markers (CD45RA, CCR7 and CD27) and compared them MM and severe individuals (**Figures 1A**, **S2A, B**). We performed multivariate statistical analysis to account for the influence of differences in age, gender, DfSO to sample, co-morbidities and treatments between groups. Analysis of CD4+ T-cell subsets revealed lower levels of CD4+ TCM and TTM in NSC compared to MM (p=0.0347, p=0.0024 **Figure 1B**). No significant differences were observed regarding the distribution of any other CD4+ T-cell subset between study groups (**Figure 1B**). Interestingly, we observed marked differences in CD8+ T-cell subsets, when comparing study groups (**Figure 1C**). Specifically, NSC had significantly higher frequencies of CD8+ T naïve (TN, median 44.9% NSC vs 33.1% MM and 18.11% Severe; p=0.0119 and p=0.0250, respectively). Similarly, we observed significantly lower frequencies of CD8+ T effector cells (TEF) as compared with MM and Severe (median 5.5% NSC vs 16.3% MM and 36% Severe; p=0.0263 and p=0.0025, respectively). The T-cell subset distribution observed in NSC was stable up to a median of 92.5 Days from Symptom Onset (DfSO) to this second sample (**Figures S2C, D**).

Then, we further investigated the association between the levels of CD8+ TN and TEF and age or DfSO to sample between groups. Meanwhile, no significant correlation between age and frequencies of CD8+ TN nor TEF was found in NSC (**Figures 1D, E**). There was a significant negative correlation between frequency of CD8+ TN and age in MM and Severe groups (Spearman r=-0.6059, p=0.0107 and r=-0.6249, p=0.0107, respectively) (**Figure 1D**) and a significant positive correlation between frequency of CD8+ TEF and age (r=0.5594, p=0.0324 and r=0.5556, p=0.0274) (**Figure 1E**) in the MM and Severe groups. Also, no significant correlations were found between DfSO to sample and CD8+ TN or TEF in any of the study groups (**Figures 1F, G**). Taken together, these data indicate a skewed CD8+ T-cell subset distribution towards high levels of naïve and low levels of effector cells independent of age in NSC.

### Non-Seroconvertors Display Lower Levels of T-Cell Activation-Induced Markers and PD-1

**Figure 2:**
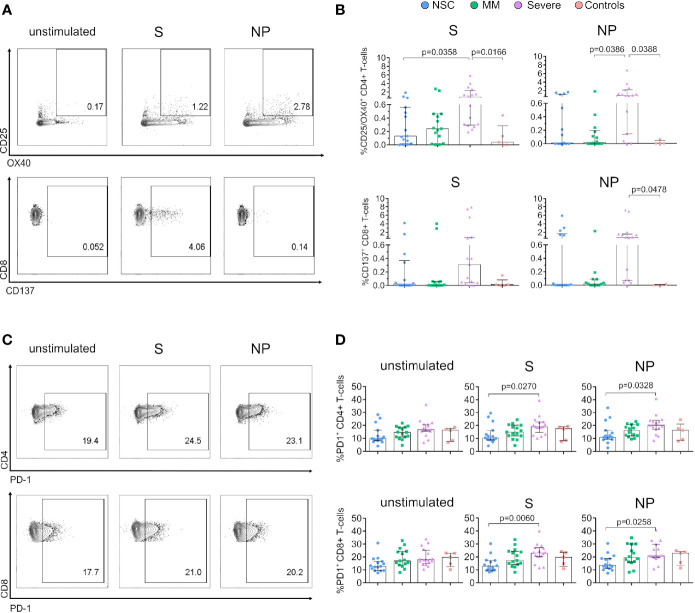
Expression of activation markers is lower in SARS-CoV-2 Non-Seroconvertors compared to seropositive individuals. PBMCs from Non-Seroconvertors and comparative study groups were stimulated for 17 hours with S and NP recombinant proteins and stained using antibodies directed against activation-induced markers (AIM; CD25, OX40, CD137) and PD-1, and analysed by flow cytometric analysis. Cells were pre-gated for lymphocytes and live cells. **(A)** Representative dot-plots of AIM expression in CD4+ and CD8+ T cells. **(B)** Bar graphs showing the expression of activation induced markers in CD4+ (CD25^+^/OX40^+^) and CD8+ T cells in response to S and NP (CD137^+^). Values from unstimulated cells were subtracted for each individual. **(C)** Representative dot-plots of PD-1 expression on CD4+ and CD8+ T cells. **(D)** Bar graphs of the expression of PD-1 on CD4+ and CD8+ T cells in unstimulated cells as well as following S and NP stimulation. Error bars represent the interquartile range and median values per group are shown. Statistical analysis was performed using Kruskal-Wallis non-parametric ANOVA adjusted for multiple comparisons, only significant differences are highlighted in the Figure.

Next, we used PD-1 and activation-induced markers (AIM) to evaluate total T cell and SARS-CoV-2-specific T cell activation status (25, 35). AIMs have been used to identify antigen-specific CD4+ T cells *via* the detection of upregulated surface markers following antigen stimulation (36). We assessed general T-cell activation and AIMs using antibodies directed against CD25, OX40 and CD137 and PD-1 in unstimulated and S- and NP-antigen stimulated conditions (**Figures 2A–C**). We observed significantly lower expression of CD25^+^/OX40^+^ CD4+ T cells in response to S in NSC compared to Severe (median 0.13% vs 0.82%, respectively, p=0.0358, **Figure 2B**). In addition, CD25^+^/OX40^+^ CD4+ T cells in response to NP were significantly lower in MM as compared to Severe (0.02% vs 0.85% p=0.0386) (**Figure 2B**). Despite the low activation, we observed CD4+ and to a lesser extent CD8+ T-cell proliferation in response to S and NP in two out of two assessed NSC (**Figure S3**), supporting the functional capacity of antigen specific T-cells.

The analysis of AIMs on CD8+ T cells did not reveal significant differences between study groups despite a lower median frequency in NP CD137^+^ CD8+ T cells between NSC and Severe (0% vs 0.78%) (**Figure 2B**). In addition, to determine potential changes in activation over time we performed a longitudinal follow-up of AIMs in six NSC individuals. As shown in **Figure S2E**, the frequencies of S and NP CD25^+^/OX40^+^ CD4+ T cells decreased or remained undetectable between time points in all the NSC except for one individual, who also displayed increased cytokine production in response to S. A comparable profile was observed for S and NP CD137^+^ CD8+ T cells over time (**Figure S2E**).

Along with decreased T-cell functionality, changes in PD-1 expression have been associated with severe COVID-19 (37). Consistently, our analysis of PD-1 demonstrated a significant reduction in the frequency of PD-1 expressing T cells in NSC compared to Severe individuals in S- and NP-stimulated conditions (**Figure 2D**) in both CD4+ (S, p=0.0270 and NP, p=0.0328) and CD8+ (S, p=0.0060, and NP, p=0.0258). Also, longitudinal follow-up revealed stable frequencies of PD-1 expression in CD4+ and CD8+ T cells in NSC a median of 95.2 days (**Figure S2F**). Only we observed a decrease in the frequency of PD-1^+^ CD4+ T cells following S-stimulation over time (p=0.0313, Wilcoxon matched-paired signed-rank test, **Figure S2F**). Collectively, these data indicate lower levels of AIMs in CD4+ T cells in response to S and low PD-1 expression in both CD4+ and CD8+ T cells in responses to S and NP SARS-CoV-2 antigens as a specific immunological trait in NSC.

### Functional SARS-CoV-2-Specific CD4+ and CD8+ T Cell Responses in Non-Seroconvertors

**Figure 3:**
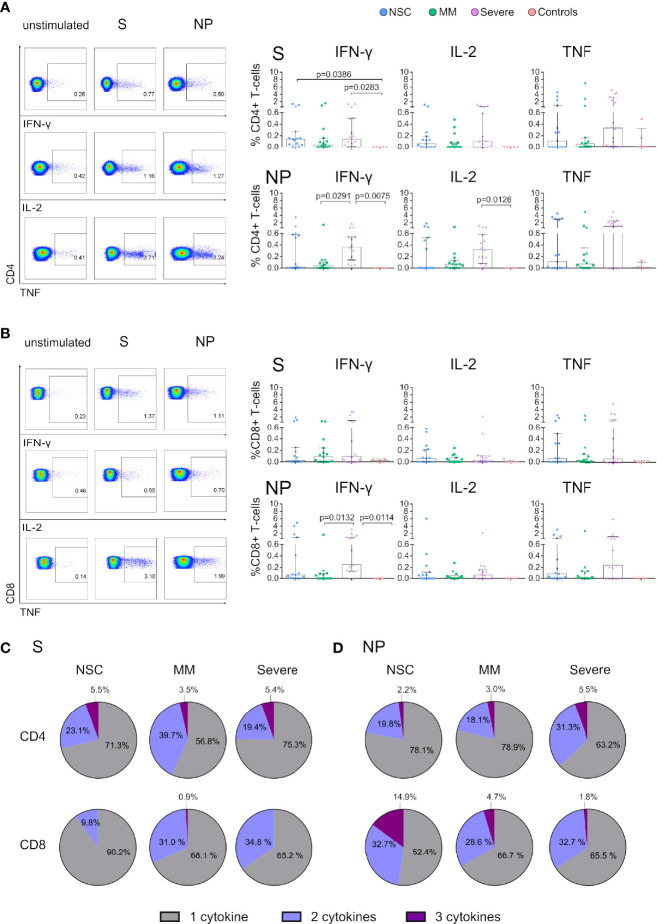
Functional T cells of SARS-CoV-2 Non-Seroconvertors are present in NSC in response to Spike and Nucleocapsid. Cryopreserved PBMCs from SARS-CoV-2 Non-Seroconvertors (NSC) and comparative study groups, Mild-moderate (MM), Severe, and controls samples were stimulated for 17 hours with Spike (S) and Nucleocapsid proteins (NP). An unstimulated condition was used as a control and values were subtracted. Cells were pre-gated for lymphocytes and live cells. Production of IFN-γ, IL-2, and TNF was monitored by Flow cytometric analysis following intracellular cytokine staining. **(A, B)** Left panels depict the gating strategy for analysis of cytokine expression and right panels represent the frequencies of IFN-γ, IL-2, and TNF in CD4+ **(A)** and CD8 **(B)** T cells in the study groups in response to S and NP. Pie charts demonstrate the relative proportions of T cells producing one, two or three cytokines in response to S in CD4+ T cells and CD8+ T cells **(C)**. Relative proportions of T cells producing one, two or three cytokines in response to NP in CD4+ T cells and CD8+ T cells **(D)**. Statistical analysis was performed using non-parametric ANOVA (Kruskal Wallis) adjusted for multiple comparisons. Error bars represent the interquartile range and median values per group are shown. Only significant differences are indicated in the Figure.

The importance of functional virus-specific T-cell responses in SARS-CoV-2 disease outcomes has been consistently reported (17, 38). Furthermore, T-cell responses against S, NP and M SARS-CoV-2 proteins have been identified in Convalescent seronegative individuals (26, 27). To evaluate SARS-CoV-2 specific T-cell responses, we stimulated PBMCs with S and NP SARS-CoV-2 proteins and performed intracellular cytokine staining for IFN-γ, IL-2 and TNF production (**Figure 3**). Analysis of S and NP-specific CD4+ T-cell responses revealed antigen-specific cytokine production in all study groups (**Figure 3A**). No significant differences were found in the frequency of S and NP-specific CD4+ T cells between NSC, MM or Severe for any of the cytokines studied. However, MM displayed significantly lower NP-specific IFN-γ production than Severe individuals (p=0.0291). We observed lower median frequency of NP-specific CD4+ T cell responses in NSC vs Severe individuals (0.11% vs 1.04% in TNF, 0.02% vs 0.36% in IFN-γ and 0% vs 0.32% in IL-2, respectively; **Figure 3A**), despite no statistical differences. Similarly, S and NP-specific CD8+ T-cell responses were present in all study groups in the absence of statistically significant differences between NSC and MM or Severe groups (**Figure 3B**). However, median frequencies of IFN-γ or TNF producing NP-specific CD8+ T cells were lower in NSC vs Severe (0.08% vs 0.24% in TNF and 0.06% vs 0.25% for IFN-γ, respectively). Significantly lower NP-specific IFN-γ production was found between MM and Severe (p=0.0132) (**Figure 3B**). Next, we evaluated the frequency of T-cell responders across study groups defined by the presence of either S or NP stimulated CD4+ or CD8+ T cells positive for one AIM or cytokine. Overall, the frequency of SARS-CoV-2 responders were 87% in NSCs, 73% in MM, 94% in Severe, and 20% in healthy controls. Distributed by SARS-CoV-2 proteins, we observed a frequency of S responders in 73% of NSCs, 73% of MM, 94% Severe, and 20% of healthy controls. The frequency of NP responders was 53% in NSCs, 50% in MM, 86% in Severe, and 0% in healthy controls. These data are consistent with previous studies demonstrating the presence of SARS-CoV-2 reactive T cells in seronegative SARS-CoV-2 convalescent individuals and 50% of unexposed individuals (24, 25, 35).

We next assessed the polyfunctionality of S and NP-specific T cells in NSCs and comparative study groups. Polyfunctionality was assessed by Boolean gating (27). Visualization of cytokine profiles in S and NP SARS-CoV-2-specific T cells was performed by UMAP analysis as represented in **Figure S4**. In response to S proteins, CD4+ T cells in NSCs displayed similar characteristics to Severe, while MM had higher frequencies of bifunctional and lower of trifunctional cells (**Figure 3C**). On the other hand, CD8+ T cells in response to S displayed higher frequencies of monofunctional cells in NSC (90.2%) and lower frequencies of bifunctional cells compared with the other two groups. Negligible frequencies in CD8+ trifunctional cells were observed in all groups (**Figure 3C**). In response to NP proteins, CD4+ T cells of NSCs displayed similar characteristics to MM whereas Severe had a higher proportion of bifunctional cells (**Figure 3D**). Interestingly, NSC had the highest frequencies of trifunctional NP-specific CD8+ T cells, accounting for 14.9% compared with MM and Severe (**Figure 3D**) and a trend toward higher trifunctional cells was observed in NSC compared to Severe (p= 0.0642, Mann-Whitney test). By contrast, no major differences in mono and bifunctional CD8+ T cells between groups were observed (**Figure 3D**). These results demonstrate the presence of functional SARS-CoV2-specific T cells responses in NSC.

## Discussion

While unprecedented advances have been made for the approval of COVID-19 vaccines with a high degree of efficacy (39–42), ensuring protection from infection and long-lasting immune protection and remain challenges for current SARS-CoV-2 vaccines. Severe COVID-19 has been related to increased age, high levels of inflammatory markers and seroconversion (28). Persistent seroconversion and higher neutralizing titers have been found consistently in hospitalized SARS-CoV-2 infected individuals (16, 43).

Non-Seroconvertors (NSC) present generally asymptomatic infection being present at a frequency of 2-17% among SARS-CoV-2 Convalescent individuals (25–27). Thus, presence of NSC support the relevance of alternative immune mechanisms to humoral responses important in limiting SARS-CoV-2 pathogenesis. While previous studies demonstrated the presence of SARS-CoV-2 specific T-cell responses in SARS-CoV-2 NSC (25, 27), and SARS-CoV-2 specific CD8+ T-cell responses have been very well characterized in a small fraction of those (26), to our knowledge a complete characterization of the NSC immune phenotype is still missing. Here, we have conducted a detailed immune characterization in SARS-CoV-2 individuals focusing in NSC and compare with MM and Severe in terms of T-cell subset distribution, activation and functionality in response to SARS-CoV-2 antigens.

In the context of the KING extension cohort, we identified a 4.5% of NSC using a very conservative approach; confirmed SARS-CoV-2 PCR positivity, double ELISA testing and sampling-time estimation to allow for seroconversion after infection. Moreover, we confirmed the true nature of NSC in our study by longitudinal clinical follow-up by the absence of SARS-CoV-2 IgG, IgM and IgA seroconversion. Of note, the NSC were enriched in mild to moderate SARS-CoV-2 infection cases and a high frequency of females. The bias of an increase in the frequency of females observed may indicate a specific trait of the NSC phenotype as suggested by previous studies (44). This observation is consistent with previous studies suggesting robust T-cell responses in females and less severe SARS-CoV-2 infection and death (45–47).

In this study, we characterized T-cell immunity in NSC and compared it with SARS-CoV-2 infected individuals with IgG seroconversion stratified by disease severity. In terms of T-cell immunophenotype, we observed a skewed distribution of CD8+ T cell subsets towards a higher frequency of naïve (TN) and lower frequency of effector (TEF) CD8+ T cells in NSC after adjusting for age, DfSO to sample, co-morbidities and comedications. We found a significant negative correlation between age and CD8+ TN in MM and Severe groups that did not hold in NSC. These findings are in line with previous observations indicating a decline in the frequencies of naïve T cells with age and the association between low levels of naïve CD8+ and increased risk of severity in COVID-19 (17, 48). The skewed distribution of CD8+ TN and TEF observed in NSC support specific characteristics associated with T-cell homeostasis in this group. Thus, the higher levels of CD8+ TN cells in NSC could be the result of a continuous thymic repopulation of the naïve compartment, as observed in HIV-1 viremic non-progressors individuals (49). High levels of CD8+ TN could also allow for rapid and efficient priming and expansion of SARS-CoV-2-specific CD8+ T cells favoring rapid viral control. In addition, the low levels of CD8+ TEF observed in NSC could partially be explained by the presence of polyfunctional CD8+ T cells, overcoming the need for a large proportion of effector cells.

We investigated the expression of AIMs in CD4+ T cells and CD8+ T cells, and PD-1 in response to S and NP antigens. Data from AIMs identified lower median frequencies of S CD25^+^/OX40^+^ CD4+ T cells and a trend towards lower CD137^+^ CD8^+^ T cells in response to NP antigen in NSC compare with Severe. The higher frequencies of AIM^+^ T cells observed in Severe are likely to detect cytokine profiles outside of IFN-γ, IL-2 and TNF studied and T regulatory cells as previously described (36). Also, the pro-inflammatory environment associated with COVID-19 severity may be involved in the increased immune activation found. Moreover, we observed decreased levels of PD-1 in CD4+ and CD8+ T cells in NSC compared to Severe following SARS-CoV-2 antigen stimulation. Elevated activation and exhaustion markers have been found in T cells of severe COVID-19 patients (37). Aside from elevated activation and exhaustion markers such as PD-1, decreased functionality has been observed in T cells from severe COVID-19 patients (37). Recently, SARS-CoV-2 specific-CD8+ T cells expressing PD-1 were shown to be functional rather than exhausted, as they also produced IFN-γ (50). Therefore, while the PD-1 expressing cells in Severe may be functional, our data suggest that T cells of NSC can exhibit functionality with lower PD-1 and AIM expression levels

Complementary to AIMs, we extended the characterization of T-cell immunity to functional SARS-CoV-2-specific CD4+ and CD8+ T-cell responses by cytokine production against S and NP antigens. Overall, we did not observe differences in cytokine production in CD4+ and CD8+ T cells in response to S and NP proteins between groups, marked interindividual variability found may account for the lack of statistical differences.

Targeting of T-cell responses toward S-, M- and NP-proteins in COVID-19 patient samples has previously been reported to be mainly uniform across disease severities (51). However, in NSC, S and NP-specific T cells appear to have a balanced profile of IFN-γ, IL-2 and TNF production, while Severe exhibits higher median frequencies of TNF producing cells, likely related to cytokine perturbations following severe disease (11–13). Although NSC displayed tendencies towards lower median frequencies of SARS-CoV-2-specific T-cell responses, we described a trend toward higher levels of IFN-γ^+^ IL-2^+^ TNF^+^ NP-specific CD8+ T cells in NSC. In agreement with our findings, CD8+ T-cells were also found to be mainly directed toward the NP (52) indicating that this type of response may be significant for long-term disease protection. Our data is also in line with previous findings showing NP-specific CD8+ T cells directed against the immunodominant B7/N105 epitope were detected at high frequency in pre-SARS-CoV-2 samples, as well as in acute and convalescent COVID-19. A predominantly naïve phenotype with high TCR plasticity was observed among these cells in agreement with the higher frequency of Naïve CD8+ found in NSC (53). These findings have additional meaning considering the worldwide expansion of SARS-CoV-2 variants of concern for vaccine efficacy (54–56) where further analyses of the quality of antigen-specific CD4+ and CD8+ T-cell responses may be of crucial importance to recognize emerging SARS-CoV-2 variants. Also, these data highlight the lower overall low expression of AIMs under the presence of functional SARS-CoV-2 T-cell responses in NSC.

Our study has some limitations. First, we found a high frequency of females in NSC studied. However, sex differences associated the phenotype may be taken into account. Females have been described to seroconvert at a lower rate than men (44). Second, we used S and NP proteins as opposed to optimal peptides for the study of antigen-specific T cells, which potentially may underestimate the frequency of SARS-CoV-2 -specific T cells. However, recombinant Spike protein has previously been used to detect antigen-specific T cells (57). Third, our functional study is limited to Th1-type cytokines and does not necessarily address the overall functionality of antigen-specific T cells including degranulation markers such as CD017a (58). Finally, the range of sampling time in NSC was higher than optimal in comparison with the other groups. However no statistically significant differences in sampling time were observed. Parameters such as DfSO to sample, age, co-morbidities etc. were accounted for in the statistical analysis of T-cell subset distribution.

Moreover, it is tempting to speculate that pre-existing T cell immunity derived from previous exposure to other coronaviruses mediate the NSC phenotype given the reports on pre-existing immunity in seronegative and or uninfected individuals (25, 26, 59). This would be in line with the generation of a rapid T-cell response in the absence of immune activation, it is also a plausible explanation of why the NSC can control SARS-CoV-2 in the absence of antibodies. Indeed, cross-reactive memory CD4+ T cells have been described between SARS-CoV-2 responses and common cold coronaviruses in unexposed individuals (24). Cross-reactivity to NP has previously been demonstrated in 30% of SARS-CoV-1 and SARS-CoV-2 unexposed individuals, and NP amino acids 101-120 shared a high degree of homology to with NP amino acids in MERS-CoV, OC43 and HKU1 (60).

Collectively, these data strength our understanding of T-cell immunity in the absence of seroconversion. Presence of skewed T-cell populations in the context of functional SARS-CoV-2 specific-T cells with low activation levels were specific traits of NSC. These findings further support the role of cellular immunity in COVID-19 control, and the value of T -cell immune monitoring in populations with low seroconversion rates in response to SARS-CoV-2 infection and vaccination.

## Data Availability Statement

The raw data supporting the conclusions of this article will be made available by the authors, without undue reservation.

## Ethics Statement

The studies involving human participants were reviewed and approved by the Ethics Committee of Hospital Germans Trias i Pujol PI-20-217. The patients/participants provided their written informed consent to participate in this study.

## Author Contributions

Conceptualization, JP, AK, MM, BC, CB, JC, and JB. Methodology, EJ-M, AK, OB-L, DO, JC, RP-C, MM, EM-C, BQ-S, IB, and RPe. Validation, MM, JC, JB, RPe, LM, EM-C, BQ-S, IB, AK, and JP. Resources, JP, BC, MM, JC, JB, RPa, LM, and AC. Formal analysis, EJ-M, AK, OB-L, and DO. Investigation, AK and JP. Data curation, MM, JB, JC, EMV, BQ-S, EJ-M, AK, OB-L, and DO. Software, DO. Visualization, AK, OB-L, DO, and JP. Writing – original draft preparation, AK and JP. Supervision, JP. Writing – review and editing, AK, JP, EJ-M, RP-C, EM-C, JB, MM, OB-L, JC, BQ-S, CB, and DO. Project administration, JP. Funding acquisition, JP and BC. All authors contributed to the article and approved the submitted version.

## Funding

This study is supported in part by grants from National Health Institute Carlos III (ISCIII) COV20/00660, PI17/000164 to JP and by the CIBER-Consorcio Centro de Investigación Biomédica en Red- (CB 2021), Instituto de Salud Carlos III, Ministerio de Ciencia e Innovación and Unión Europea – NextGenerationEU. The funders had no role in study design, data collection and analysis, the decision to publish or drafting of the manuscript. OB-L was supported by the grant for Catalan Government and the European Social Fund AGAUR-FI_B 00582 Ph.D. fellowship. This study has received partial funding from Grifols and the crowdfunding initiatives “https://www.yomecorono.com”, BonPreu/Esclat and Correos. The funder was not involved in the study design, collection, analysis, interpretation of data, the writing of this article or the decision to submit it for publication. We thank “CERCA Programme/Generalitat de Catalunya” for institutional support and the Foundation Dormeur for financial support.

## Conflict of Interest

The authors declare that the research was conducted in the absence of any commercial or financial relationships that could be construed as a potential conflict of interest.

## Publisher’s Note

All claims expressed in this article are solely those of the authors and do not necessarily represent those of their affiliated organizations, or those of the publisher, the editors and the reviewers. Any product that may be evaluated in this article, or claim that may be made by its manufacturer, is not guaranteed or endorsed by the publisher.

## Supplementary Material

Supplementary Figure 1 Gating strategy and control FMOs for the detection S and NP CD4+ and CD8+ T cells. **(A)** Gating strategy for CD4+ and CD8+ T cells, **(B)** Representative dot-plots summarizing the FMO strategy in CD4+ and CD8+ T cells for TNF, IFN-γ and IL-2 staining under several conditions FMO unstimulated, FMO in the presence of the positive control (SEB) and cells in the presence of antibodies and the positive control.

Supplementary Figure 2 Longitudinal evolution of T-cell subsets and antigen specific responses in SARS-CoV-2 Non-Seroconvertors. T-cell markers were assessed in two sampling timepoints in Non-Seroconvertors. **(A)** Gating strategy for differentiation of subsets in CD4+ T cells, **(B)** and CD8+ T cells. Changes in T-cell subset distributions are represented by pie charts showing CD4+ and CD8+ T-cell subset distribution at sampling timepoint 1 and 2 (TP1 and TP2) **(C)** and graphs representing the normalized subset values within phenotypes **(D)**. Differences in Cytokine production and activation induced markers between TP1 and TP2 in response to S and NP are shown in **(E)**, and PD-1 expression in CD4+ and CD8+ T cells in unstimulated, S- and NP-stimulated cells at TP1 and TP2 are shown in **(F)**.

Supplementary Figure 3 Proliferation in CD4+ and CD8+ T cells of Non-seroconvertors and seropositive individuals with severe disease. **(A)** Representative dot-plots summarizing CFSE loss in CD4+ and CD8+ T cells following stimulation with S and NP SARS-CoV-2 proteins in two NSC. Frequency of proliferating cells in NSC and seropositve individuals in response to S and NP antigens. Values from unstimulated cells were subtracted for each individual **(B)**.

Supplementary Figure 4 Cytokine expression in CD4+ and CD8+ T cells of Non-seroconvertors and seropositive individuals with severe disease. Single-cell analysis based on antigen expression (TNF, IL-2 and IFN-γ intensity) using UMAP dimensionality reduction in CD4+ T-cells following stimulation with S **(A)** and NP **(B)** as well as CD8+ T cells stimulated with S **(C)** and NP **(D)**.
