# Supplementary figures and images for: Skewed Cellular Distribution and Low Activation of Functional T-Cell Responses in SARS-CoV-2 Non-Seroconvertors
Source: Front Immunol. 2022 May 10;13:815041. doi: 10.3389/fimmu.2022.815041 (PMC9128381; doi:10.3389/fimmu.2022.815041)

Figure S1

A.

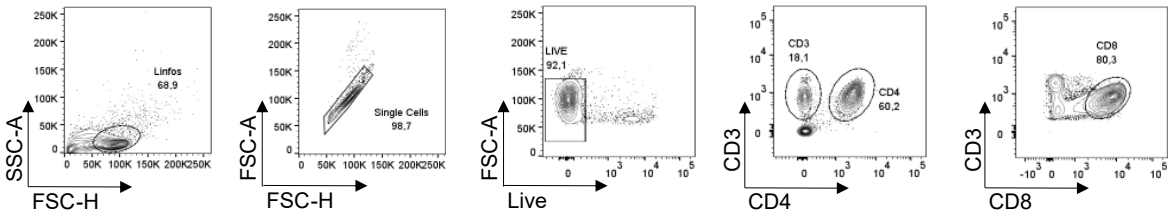

B.

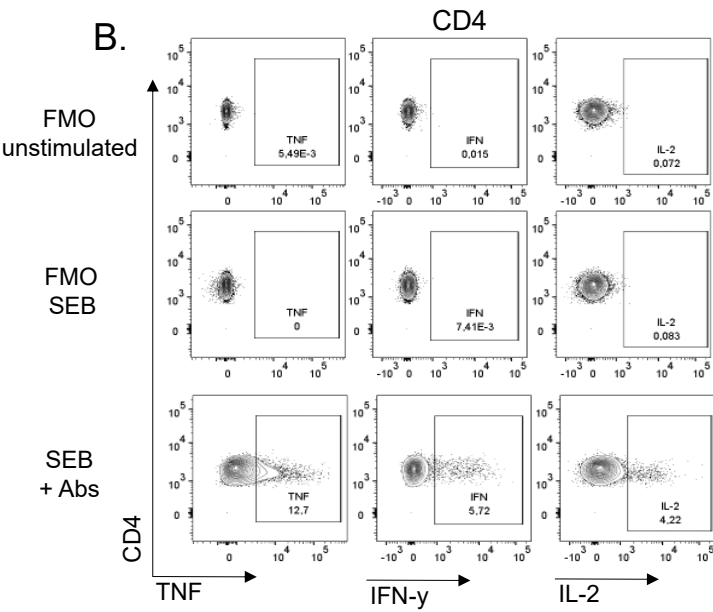

C.

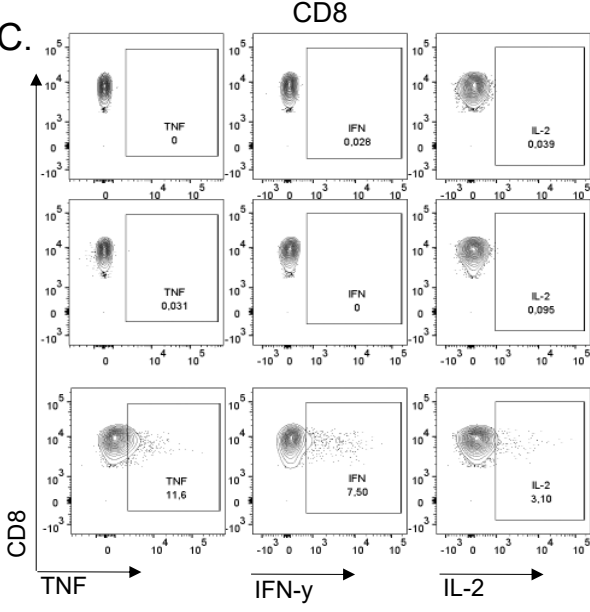

Figure S2

A.

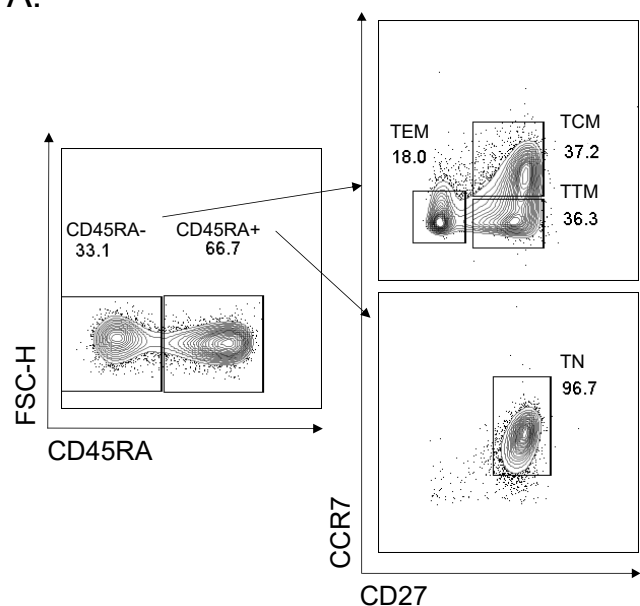

B.

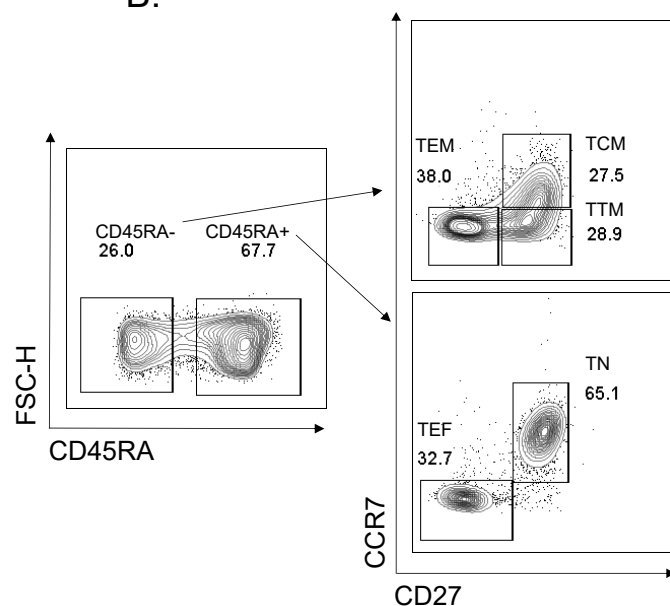

C.

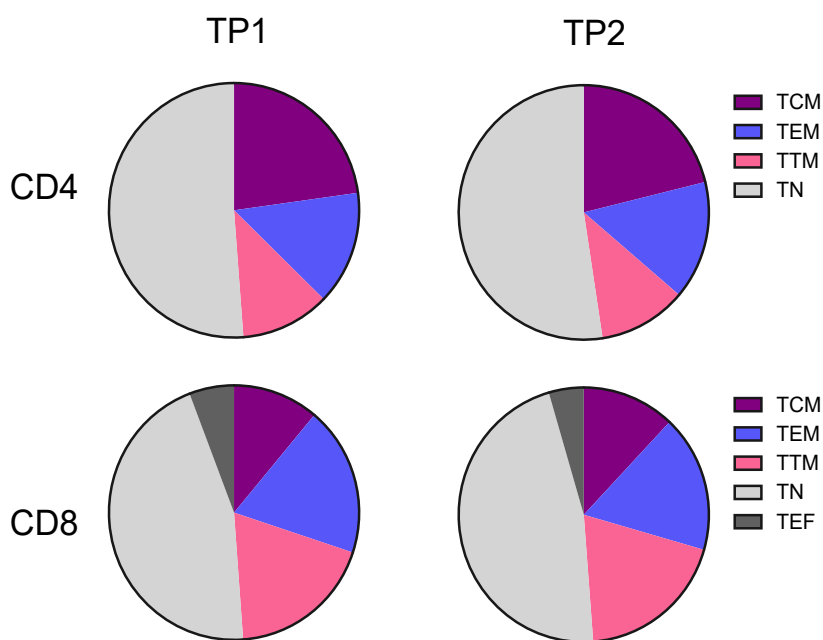

D.

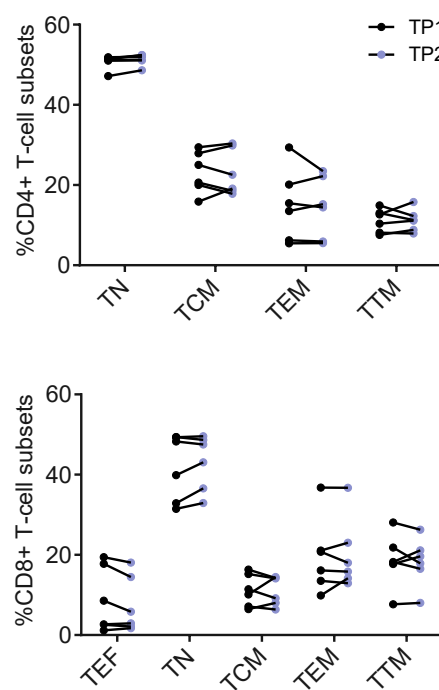

E.

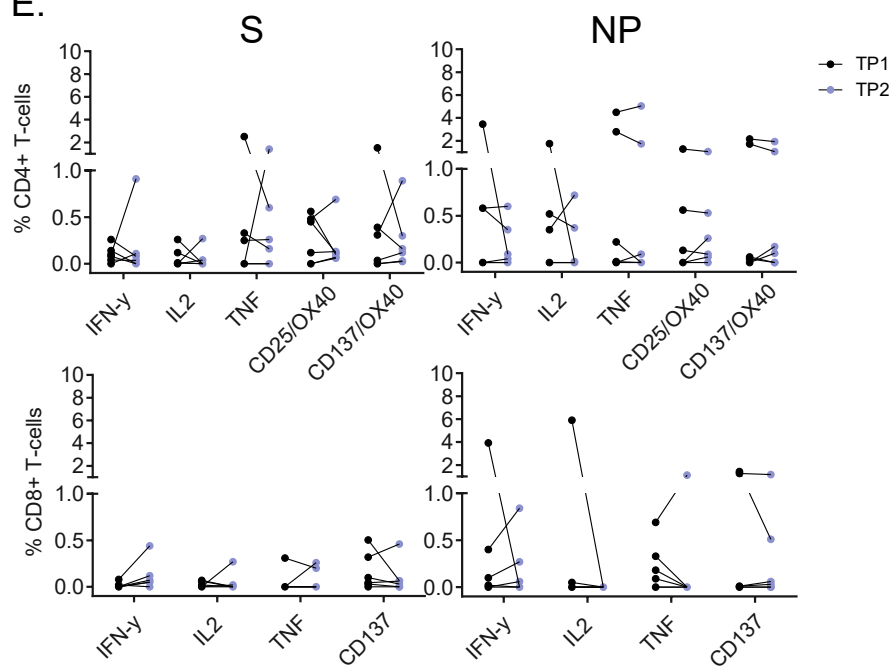

F.

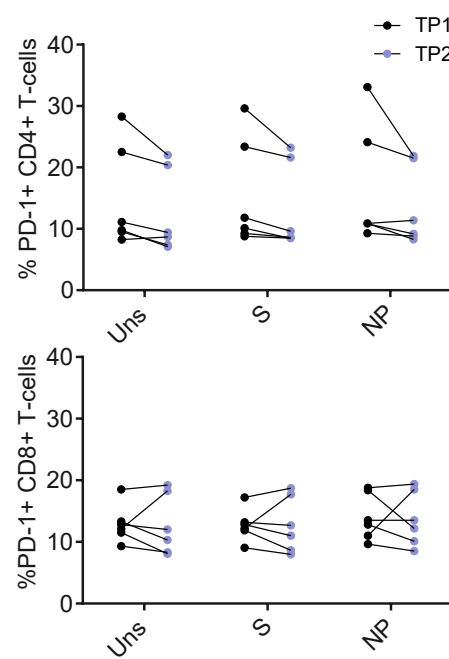

Figure S3

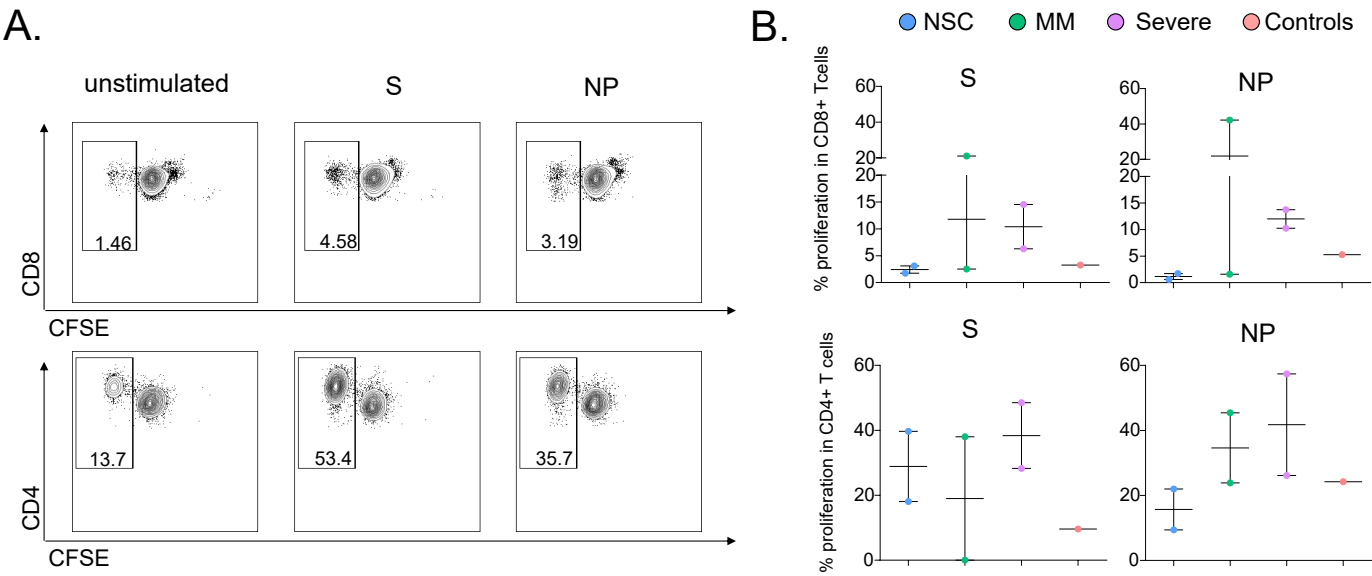

Figure S4

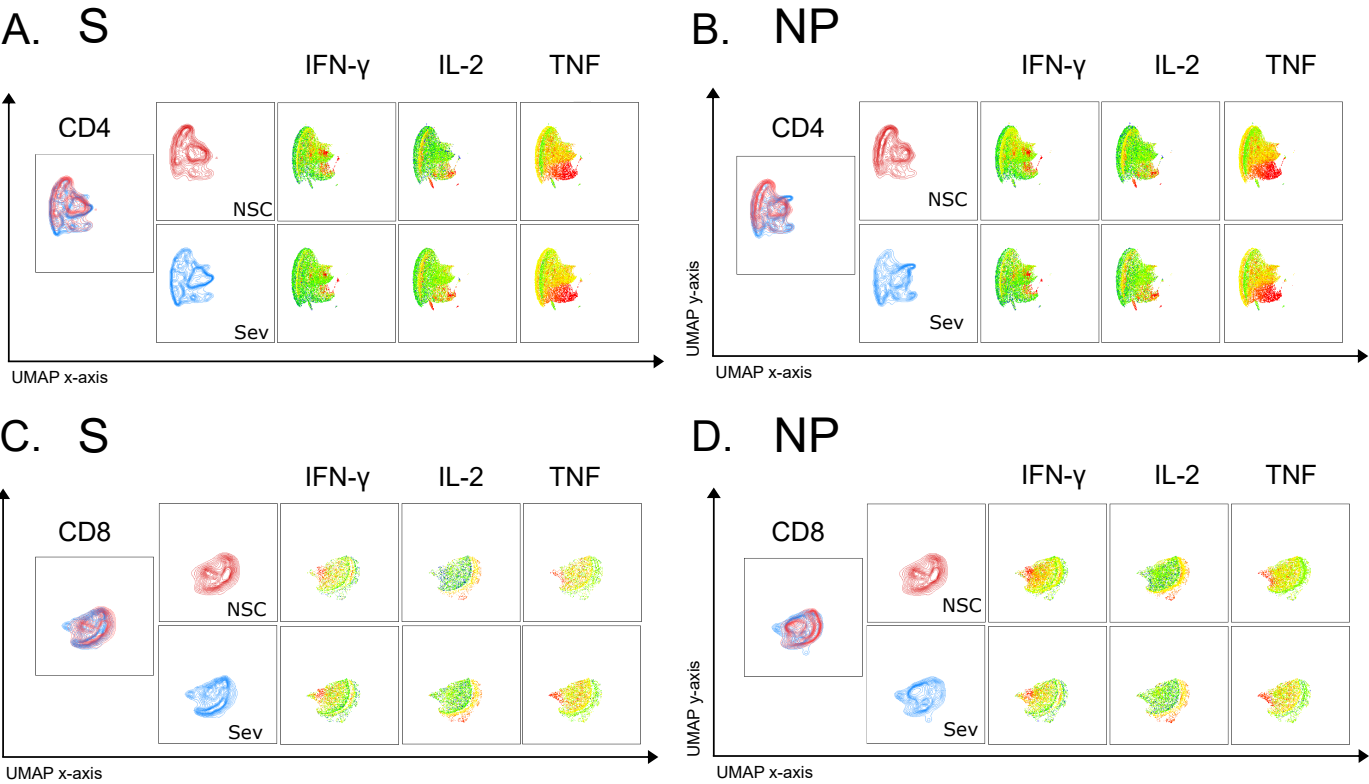

Supplement: Supplementary Figure 1 — Gating strategy and control FMOs for the detection S and NP CD4+ and CD8+ T cells. (A) Gating strategy for CD4+ and CD8+ T cells, (B) Representative dot-plots summarizing the FMO strategy in CD4+ and CD8+ T cells for TNF, IFN-γ and IL-2 staining under several conditions FMO unstimulated, FMO in the presence of the positive control (SEB) and cells in the presence of antibodies and the positive control. [file DataSheet_1.pdf]
